# Rapid reconstruction of infectious bronchitis virus expressing fluorescent protein from its nsp2 gene based on transformation-associated recombination platform

**DOI:** 10.1128/jvi.00535-25

**Published:** 2025-06-05

**Authors:** Yingfei Li, Linqing Duan, Lihua Tang, Min Huang, Ye Zhao, Guozhong Zhang, Jing Zhao

**Affiliations:** 1 National Key Laboratory of Veterinary Public Health Security, College of Veterinary Medicine, China Agricultural University 630101 , Beijing, China; 2 Key Laboratory of Animal Epidemiology of the Ministry of Agriculture, College of Veterinary Medicine, China Agricultural University 630101 , Beijing, China; University of North Carolina at Chapel Hill, Chapel Hill, North Carolina, USA

**Keywords:** reverse genetics, IBV, TAR, highly efficient, nsp2, reporter virus

## Abstract

Traditional reverse genetics strategies for large-genome RNA viruses are hindered by multiple technical bottlenecks, including dependence on specific restriction enzyme sites, cumbersome multi-step cloning, and genetic instability of oversized DNA in bacterial systems. Herein, we established a universal reverse genetics platform for infectious bronchitis virus (IBV) through systematic optimization of the transformation-associated recombination (TAR) technology. By dividing the IBV genome into seven overlapping fragments and employing *Saccharomyces cerevisiae* for seamless assembly, we obtained a stable full-length genome clone with an efficiency exceeding 95%. Optimizing cultivation temperature and selecting appropriate *Escherichia coli* strains were key improvements that minimized mutagenesis during bacterial replication, ensuring fidelity of the constructs. The rescued QX-type IBV strain replicated and induced pathogenicity in chicken embryos comparably to clinical isolates, while retaining engineered markers without additional mutations. The platform’s feasibility was further confirmed by successfully rescuing the Mass-type IBV strain, demonstrating its broad applicability. Notably, we pioneeringly rescued a reporter virus expressing the mNeonGreen fluorescent protein, linked via the porcine teschovirus 1 2A proteolytic cleavage site, immediately upstream of the IBV nsp2 gene. This design enabled autonomous separation of the reporter from viral polyproteins without the deletion of any viral gene. The recombinant virus stably maintained this insertion for at least 10 passages, marking the nsp2 site as a flexible locus for foreign gene accommodation in IBV. This study not only establishes a universal TAR-based reverse genetics platform for gamma-coronaviruses but also provides a powerful tool for visualization-based high-throughput antiviral drug screening.

**IMPORTANCE:**

Traditional reverse genetics systems for infectious bronchitis virus (IBV) are often hindered by assembly difficulties *in vitro* and viral genome instability during bacterial propagation. Here, we developed a transformation-associated recombination-based platform for seamless IBV genome assembly and rapid virus rescue within 12 days. Additionally, we identified a novel foreign gene insertion site between the 5′ UTR and nsp2 in the viral genome, enabling stable fluorescent protein expression without deleting any viral genes, ensuring that virus replication is not affected. This system provides a powerful tool for tracking IBV infection, studying viral tropism, and screening antivirals, thereby advancing coronavirus research and poultry disease control.

## INTRODUCTION

Coronaviruses have emerged as pivotal agents of public health crises over recent decades, epitomized by the 2003 SARS-CoV outbreak, the 2012 MERS-CoV epidemic, and the SARS-CoV-2 pandemic initiating in late 2019, all of which have exerted unparalleled strain on global healthcare systems and incurred profound socioeconomic disruptions (1, 2). These coronavirus-mediated diseases have posed unprecedented challenges to global health systems and caused substantial socioeconomic impacts worldwide. Within the *Coronaviridae* family, the Coronavirinae subfamily represents a genetically diverse group of viruses classified into four distinct genera (*Alphacoronavirus, Betacoronavirus, Gammacoronavirus*, and *Deltacoronavirus*) based on their genomic characteristics (3). As a key gammacoronavirus, avian infectious bronchitis virus (IBV) induces a multisystemic pathology in chickens. The diverse tissue tropism of different IBV genotypes contributes to the complexity of clinical manifestations. Clinical symptoms include sneezing, coughing, tracheal rales, and nasal discharge after respiratory infection. Infection of the urinary system results in pale, swollen kidneys and urate deposition, leading to weight loss and increased mortality. Infected female chickens experience abnormal oviduct development, leading to a sharp decline in egg production during the laying period, which subsequently impacts production efficiency (4, 5).

IBV is an enveloped, single-stranded, positive-sense RNA virus, with a total genome length of approximately 27.7 kb that encodes four structural proteins, namely spike protein (S), accessory proteins 3a and 3b, small envelope protein (E), membrane protein (M), accessory proteins 5a and 5b, and nucleocapsid protein (N) (6). Approximately two-thirds of the full-length genome at the 5′-end encodes two replicase proteins (1a and 1b), which are further processed to form 15 non-structural proteins (nsp2–16) (7). According to the amino acid sequence of full-length S1, IBV can be divided into nine genotypes (GI-GIX), with dozens of genetic lineages (8). In farms, GI-19 (QX-type) accounted for the highest proportion, up to 70%, followed by GI-13 (793/B-type, also known as 4/91) and G1-7 (TW-I). Since 2007, the prevalence of GVI-1 (TC07-2) strains in chickens has steadily increased, but the pathogenicity is less serious. Currently, control of IBV primarily relies on live attenuated and inactivated vaccines. However, the genetic diversity of IBV necessitates continuous vaccine updates, posing challenges for disease prevention and control (4, 6, 8, 9).

Although the etiological characteristics of IBV—such as broad tissue tropism (10), frequent antigenic drift (11), and the co-circulation of multiple genotypes (12)—have been well documented, the molecular basis underlying its key virulence determinants and immune evasion mechanisms remains poorly defined. Conventional phenotype-genotype correlation studies relying on field isolates often fail to dissect the functional consequences of individual mutations within a controlled genetic context. To address this, reverse genetics, which enables precise genome editing and recovery of recombinant viruses, has emerged as a pivotal tool for deciphering genotype-phenotype relationships in IBV. The reverse genetics for IBV has progressively evolved, primarily relying on two established methodologies: *in vitro* ligation and bacterial artificial chromosome (BAC)-based systems. The *in vitro* ligation strategy exploits IIS restriction enzymes, which recognize variable sequences without introducing extraneous restriction sites. Purified genomic fragments are assembled into full-length cDNA *in vitro*, followed by T7 RNA polymerase-driven transcription and electroporation of synthesized RNA into permissive cells for viral rescue. While this strategy circumvents bacterial replication-associated mutagenesis, its dependence on multi-fragment assembly renders the process labor-intensive and technically demanding (13–15). In contrast, the BAC system employs low-copy mini-F plasmids to mitigate the cytotoxicity of coronavirus genomes in *Escherichia coli*, thereby enhancing replication fidelity (16–19). However, this method necessitates the utilization of intrinsic restriction sites or the introduction of silent mutations to facilitate the cloning of the IBV genome into BAC-compatible fragments. The original IBV reverse genetics system was developed using vaccinia virus as the vector, which leveraged its large genomic capacity to incorporate foreign genes and maintained high fidelity replication to preserve genetic stability. Nevertheless, due to its technical complexity, time-consuming procedures (20, 21), and reliance on homologous recombination within eukaryotic cells, which is difficult to control, this method has been phased out. A more specialized approach, targeted RNA recombination, exploits the inherent recombination propensity of RNA viruses but is limited to modifying the 3′ terminal third of the genome, severely restricting its broader applicability (22). A paradigm shift emerged during the SARS-CoV-2 pandemic with the advent of yeast homologous recombination technology, which enables rapid (<14 days) and seamless assembly of full-length coronavirus genomes (23). The underlying principle of employing the yeast cloning system is the ability of yeast to recombine overlapping DNA fragments, which is called transformation-associated recombination (TAR) (24). This platform overcomes the technical barriers in reverse genetic manipulation of large RNA viral genomes through traditional approaches (25). Despite its positive impact on betacoronavirus research, the practicality of this yeast-based strategy to avian coronaviruses—particularly those with distinct genotypes—remains unexplored.

The primary objective of this study focused on the evaluation of the TAR cloning system’s universality through its application to different genotypes of IBV, addressing whether its advantages in rapid assembly extend to non-mammalian coronaviruses. By cloning full-length genomes of QX and Massachusetts serotype IBV into YAC-BAC shuttle vectors, coupled with the optimization of cultivation temperature and *E. coli* strains, we improved plasmid replication fidelity. Recombinant viruses were successfully rescued following transfection of BHK-21 cells and subsequent propagation in chicken embryos. The parental and recovered viruses had nearly identical phenotypes in infected cells. The second goal was to screen a permissible insertion site within the IBV replicase 1a gene that allows the expression of a heterologous fluorescent protein without deleting any viral genes. We found that recombinant viruses with the fluorescent gene inserted upstream of nsp2 could be successfully rescued and exhibited sufficient genetic stability. Our data demonstrate that the established reverse genetics platform and the generated reporter viruses serve as powerful tools for monitoring IBV replication, cell tropism, viral transmission, and pathogenesis.

## RESULTS

### Construction of the infectious cDNA clone of IBV strains SD

**Fig 1:**
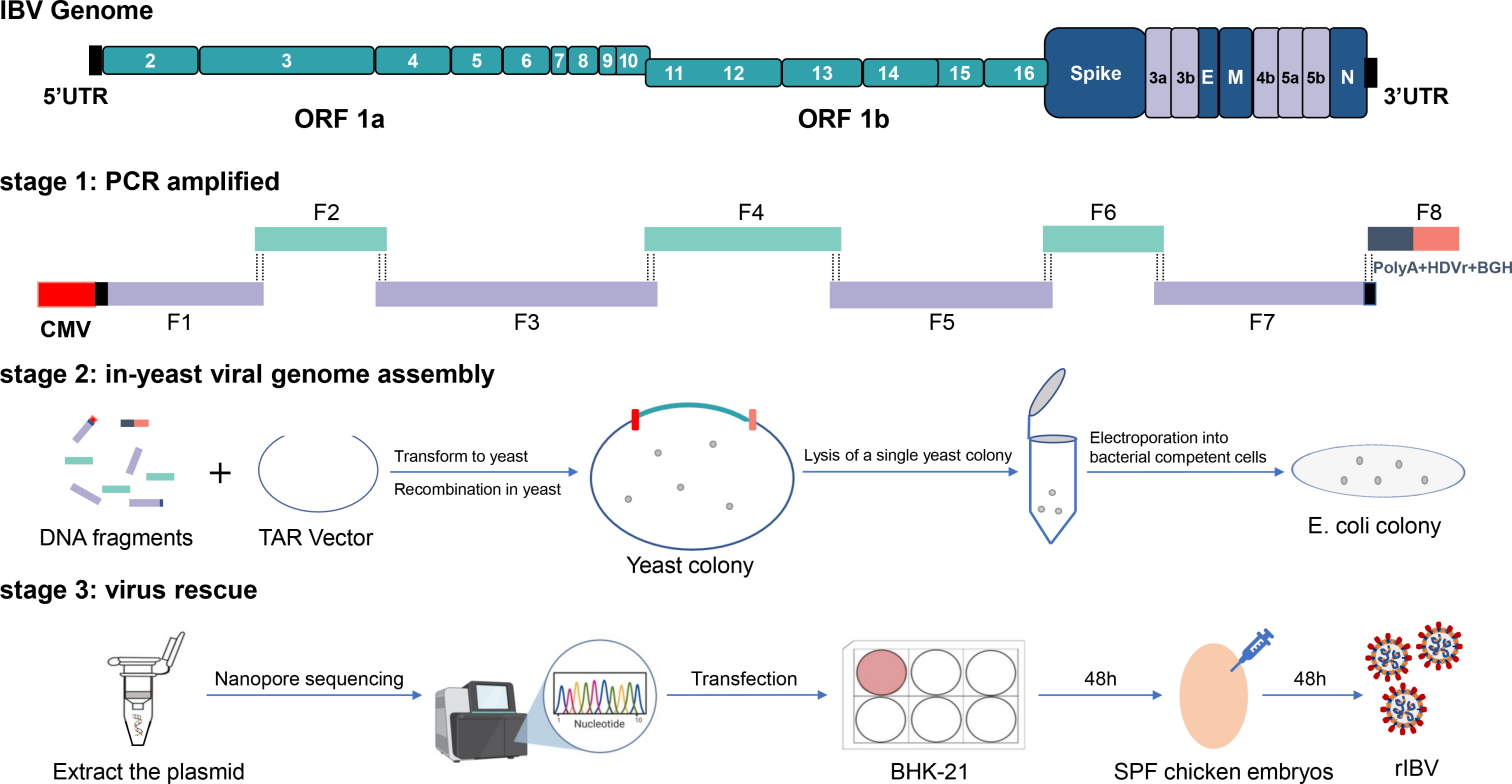
Schematic representation of TAR cloning-based reverse genetics systems for constructing an IBV cDNA clone. Relevant sequences are indicated. UTR, untranslated regions; ORF, open reading frame; the IBV genome is divided into several overlapping cDNA fragments (F1–F7). The first fragment contains overlapping sequences of the TAR vector pYES1L and a CMV (cytomegalovirus) promoter at the 5′ end. The clone was flanked at the 3′ end by the HDV ribozyme and BGH termination and polyadenylation sequences. SPF, specific pathogen free.

**Fig 2:**
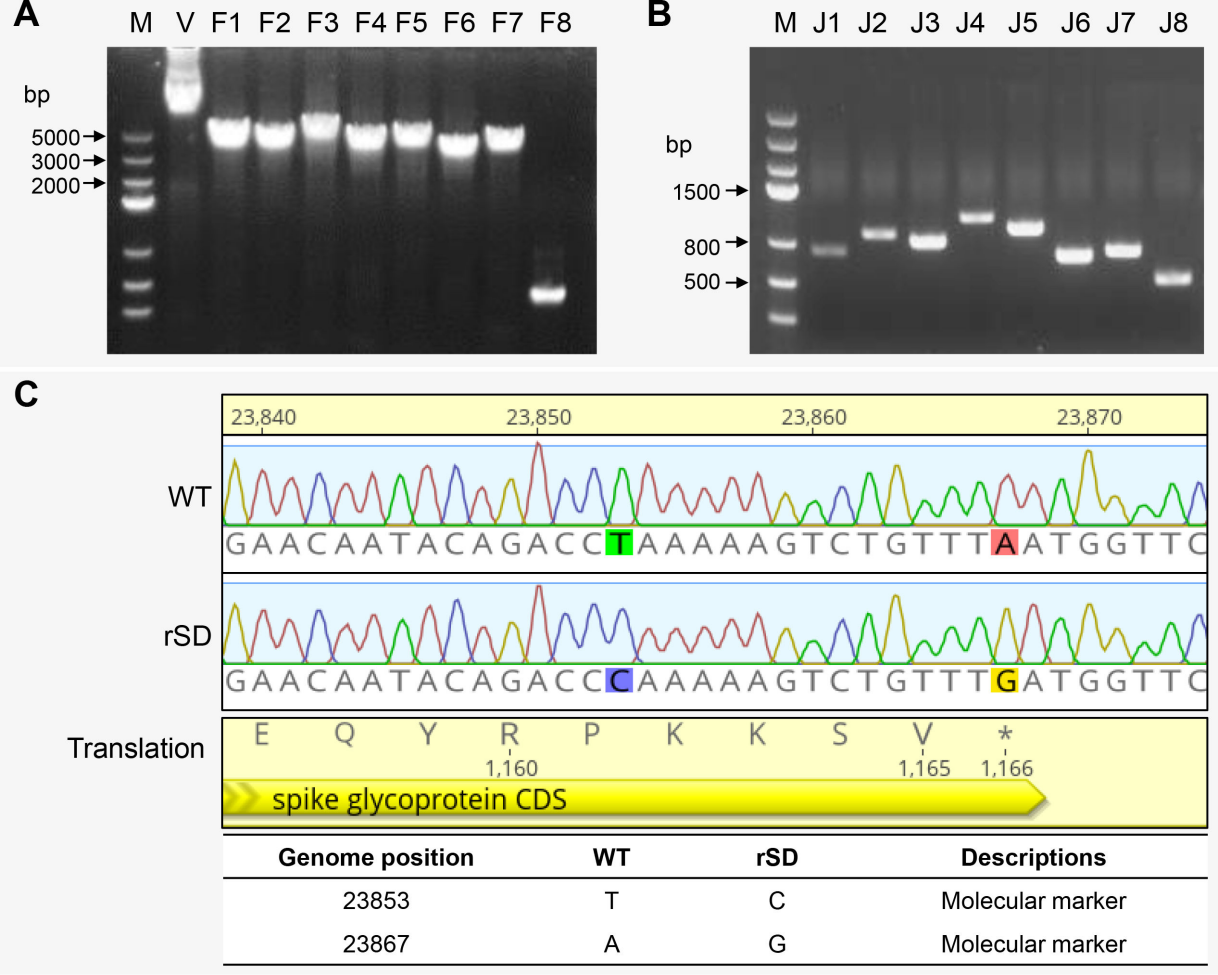
Assembled fragment preparation and verification. (**A**) Division of IBV full-length genome into seven equally sized fragments via PCR, each containing 30–50 bp overlaps between adjacent fragments (V, linearized vector and F, fragment). (**B**) Validation of yeast assembly at all fragment junctions using primers spanning adjacent regions (J, junction site). (**C**) Sequence differences between the original clinical isolate SD (WT) and the recombinant P1 rSD. The two silent nucleotide changes were engineered as molecular markers. Chromatograms of Sanger sequencing results. The engineered molecular maker mutations are indicated.

To bypass *in vitro* transcription, the infectious clone was engineered to directly generate genomic RNA transcripts within eukaryotic cells. The full-length SD genome was partitioned into seven overlapping fragments flanked by 40 bp homologous arms (F1–F7), driven by the human cytomegalovirus (CMV) immediate-early promoter. Transcription was terminated precisely at the bovine growth hormone (BGH) polyadenylation (F8). Furthermore, a hepatitis delta virus (HDV) ribozyme sequence was incorporated at the 3′ terminus of the viral genome, enabling self-cleavage of non-viral sequences to ensure the production of authentic IBV genomic RNA with resolved termini (18). The overall strategy is shown in Fig. 1. All DNA fragments were generated by PCR (Fig. 2A), and all fragments were simultaneously transformed into *Saccharomyces cerevisiae*, and PCR was used to screen for the correctly assembled yeast artificial chromosome (YAC) containing the cloned SD genome. The PCR primers were designed to cover the junctions between all recombined fragments (Fig. 2B). Since yeast culture requires specific experimental conditions and yeast cells have a thick cell wall, direct plasmid extraction is inefficient (data not shown). The pYES1L vector used in this study is a shuttle vector, with oriT allowing it to carry large genomes and replicate in bacteria. After one-step assembly in yeast, single yeast cells were lysed to release the YAC, which was then electroporated into *Escherichia coli*-competent cells for further plasmid amplification. Purified YAC-BAC shuttle plasmids were co-transfected into BHK-21 cells with pCMV-IBV-N, encoding the nucleocapsid protein of the IBV SD strain. At 48 h post-transfection, the transfected cells were inoculated into 10-day-old specific pathogen-free (SPF) chicken embryos. To genetically differentiate rescued virus from wild-type (WT), two synonymous mutations (T23853C and A23867G) were synthetically introduced into the viral spike (S) gene, confirmed by Sanger sequencing (Fig. 2C).

**Fig 3:**
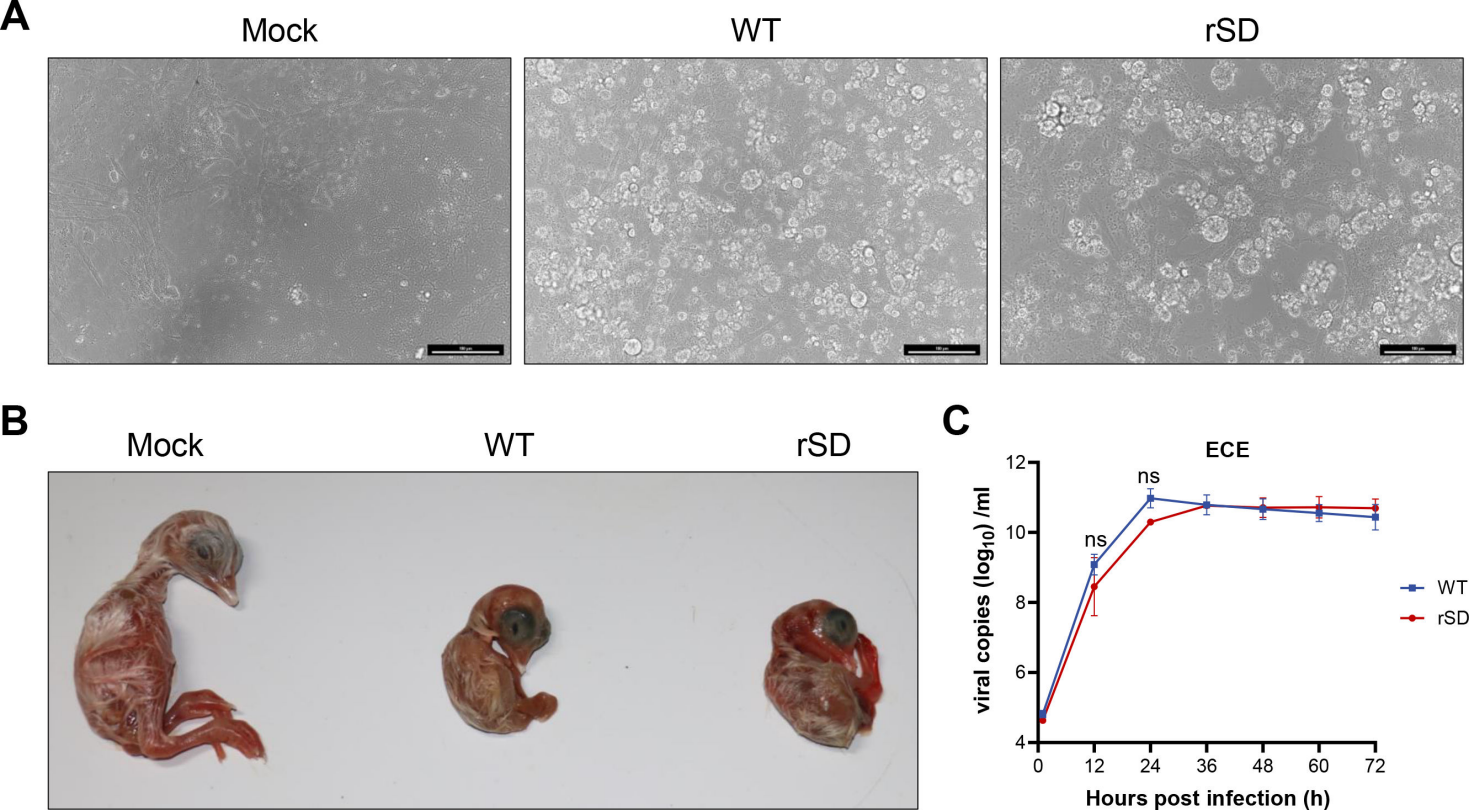
Biological characteristics between parental and recovered viruses. (**A**) Cytopathic effects observed in CEK cells under bright-field images at 48 h post-infection (hpi) with the virus (multiplicity of infection = 0.1). (**B**) Embryo lesions (curling, stunting, and dwarfing) in ECE inoculated with P2 IBV-rSD and WT. (**C**) Comparison of the replication kinetics of IBV-rSD and WT in ovo. The rescued virus IBV-rSD and parental virus (0.2 mL of 102 copies) were inoculated into the allantoic cavities of 10-day-old embryonated eggs, and the allantoic fluid of five eggs from each group was harvested at the time points 1, 12, 24, 36, 48, 60, and 72 hpi, and the viral copies were determined using RT-qPCR. Data represent the mean ± sd of three independent biological experiments (*n* = 3). Statistical significance was determined by two-sided unpaired Student’s *t*-test without adjustments for multiple comparisons. NS, not significant.

The rescued virus (rSD) exhibited the same cytopathic effects as the wild-type virus 48 h after infecting chicken embryo kidney cells (Fig. 3A). Similarly, after 144 h of infection in chicken embryos, it induced characteristic embryo curling comparable to those caused by the wild-type virus (Fig. 3B). Finally, we assessed the replication kinetics of the recovered viruses, which were indistinguishable from the parental strain, confirming successful recovery of biologically functional progeny virions (Fig. 3C). Collectively, the results demonstrate that the TAR-based technology is capable of rescuing recombinant viruses that recapitulate the replication properties of the original clinical isolate (SD-WT) in chicken embryos and chicken embryo kidney cells.

### Optimization of TAR workflow for high-fidelity assembly of the IBV genome

**Fig 4:**
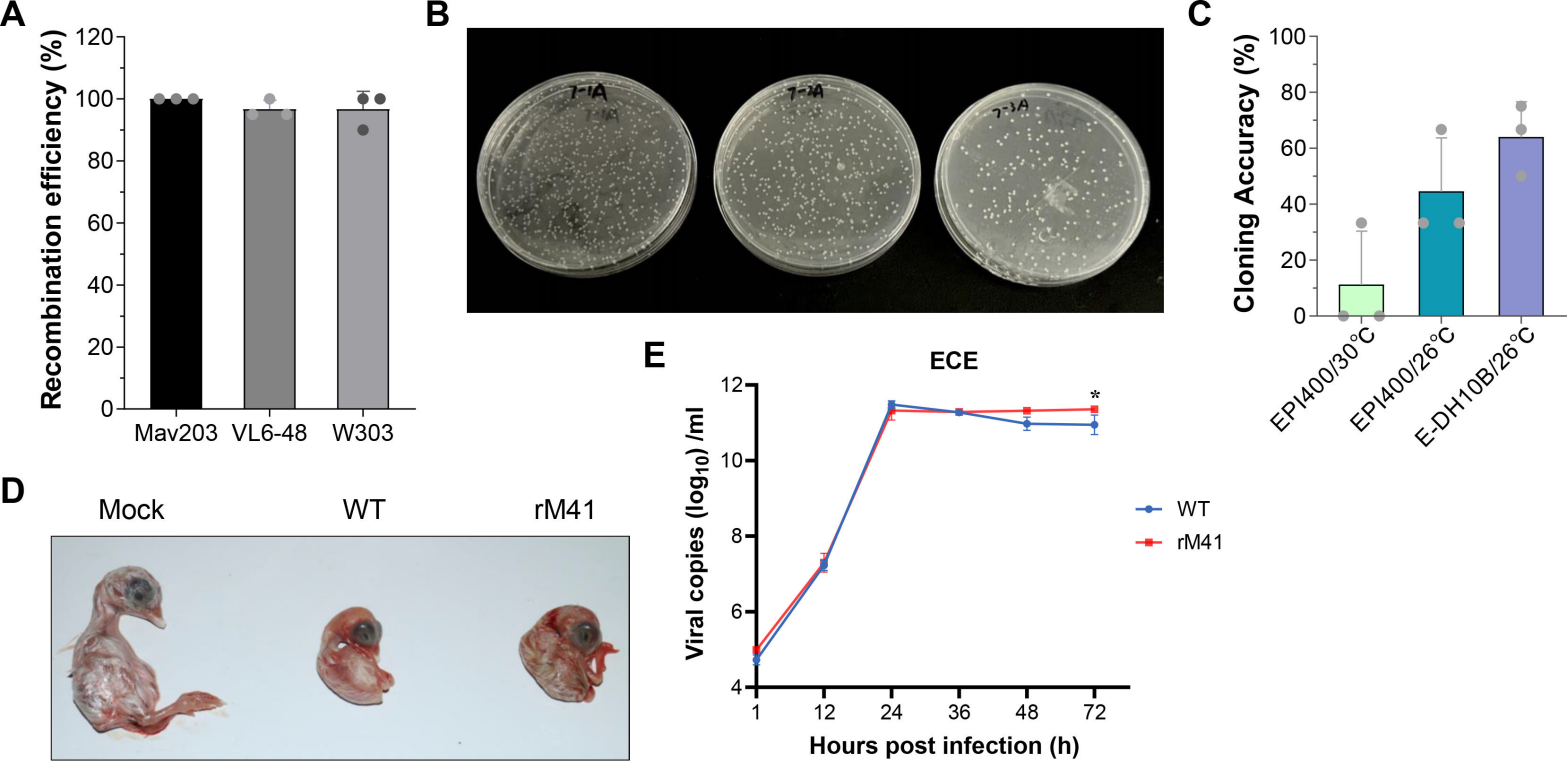
Optimization of the virus rescue protocol. (**A**) Recombinational efficiency analysis of different yeast strains. Fragments were transformed into three *S*. *cerevisiae* strains; 100 randomly selected single colonies were analyzed per replicate (*n* = 3 biological replicates), and recombination success was validated by insert junction PCR amplification. (**B**) Yeast cell wall disruption using lysis buffer. Optimal recombinant yeast strain was lysed with lysis buffer (1 M sorbitol, 50 mM Tris, 10 mM EDTA, and 1% SDS, pH 7.5). (**C**) The impact of different temperatures and strains of *E. coli* on cloning accuracy. Nine single bacterial colonies were randomly selected for each experiment to verify whether there are mutations through sequencing. Results from triplicate experiments were presented with error bars indicating standard deviations. (**D**) Embryo lesions (curling, stunting, and dwarfing) in ECE inoculated with P2 IBV-rW41 and WT. (**E**) Comparison of replication curves of IBV-rM41 and WT-M41 in chicken embryos. Allantoic fluid was collected at specified time points (1, 12, 24, 36, 48, and 72 hpi) for viral load estimation via RT-qPCR. Data represent the mean ± sd of three independent biological experiments (*n* = 3). Statistical significance was determined by two-sided unpaired Student’s *t*-test without adjustments for multiple comparisons. *P = 0.0176.

We systematically optimized key steps of the TAR workflow to further enhance the advantages of yeast-based assembly while minimizing mutations introduced during bacterial replication (Table 1). The specific procedures are as follows: comparative analysis of *S. cerevisiae* strains (MAV203, VL6-48, and W303) revealed robust genome assembly efficiencies of 100%, 96.6%, and 96.6%, respectively, with MAV203 being selected for further experiments (Fig. 4A). A yeast lysate was developed (1 M sorbitol, 50 mM Tris, 10 mM EDTA, and 1% SDS, heated at 50°C to facilitate cell wall dissolution, followed by pH adjustment to 7.5) to address the recalcitrant cell wall integrity of *S. cerevisiae*. The lysed yeast cells are directly electroporated into *E. coli*, resulting in a large number of single colonies (Fig. 4B). The fidelity of the YAC plasmid is critical for the successful rescue of the virus. To minimize additional mutations during the amplification of YAC shuttle plasmids in *E. coli*, we compared various temperatures and *E. coli* strains. Adjusting the temperature from 30°C to 26°C increased the plasmid accuracy by 33%. Compared to using EPI400 electrocompetent cells, switching the *E. coli* strain to DH10B achieved a correctness rate of 63.89% (Fig. 4C). These results indicate that selecting the yeast strain MAV203, assembling the full-length IBV genome, directly lysing the yeast cells, electroporating into DH10B competent cells, and culturing at 26°C is an effective optimization strategy.

**TABLE 1:** Comparison of different rescue strategies for infectious bronchitis virus

Construction strategies	Advantages	Disadvantages
BAC (17)	Direct transfection of BAC plasmids Allowing the insertion of large DNA	Time-consuming Bacterial replication may lead to additional mutations Dependent on specific restriction enzyme sites
*In vitro* ligation (Golden Gate Assembly) (13)	Avoids cloning instability Type IIS restriction enzymes, with no sequence constraint	Technical challenges (low efficiency of RNA electroporation, poor stability of prepared RNA transcripts) High cost of reagents
Targeted RNA recombination (22)	Allows targeted research on the function of the S gene	Only the last third of the IBV genome can be modified There is a risk of cross-species transmission
Vaccinia virus vectors (21)	IBV genome can replicate stably in the vaccinia virus without mutation	Time-consuming Process-intensive
TAR cloning (23)	One-step cloning for assembling full-length genomes Convenient to modify any fragment of the virus Rapid virus rescue	Requires whole-genome sequencing

### Construction of M41 reverse genetics based on the optimized TAR platform

To address whether the optimized TAR-based synthetic genomics platform can be applied to other genotypes of IBV, we used a molecular YAC clone of the M41 strain, which differs from the QX type SD strain described above. M41 is a classic strain within the Mass genotype and holds significant representativeness. It is also one of the earliest IBV genotypes discovered in the poultry industry to date (26). The procedure followed the same steps as described above, but it is more convenient and time-efficient, requiring only about 12 days to rescue the infectious virus. The streamlined workflow is as follows: genome assembly (day 0)—yeast single-colony verification (day 2)—bacterial single-colony selection (day 4)—plasmid extraction (day 6)—sequencing and transfection (day 8)—chicken embryo inoculation (day 10)—recovery of infectious viral particles (day 12). Compared to the parental M41 strain, the rescued rM41 virus induced identical pathognomonic lesions of embryo stunting and curling following inoculation into SPF chicken embryos (Fig. 4D) and exhibited equivalent replication kinetics (Fig. 4E). The above results demonstrate that the reverse genetics platform based on TAR technology successfully rescued both the currently prevalent QX type representative SD strain and the classical M41 strain, demonstrating that this technology has a certain degree of universality. This provides a valuable tool for elucidating the pathogenic mechanisms of IBV.

### Development of mNeonGreen IBV

**Fig 5:**
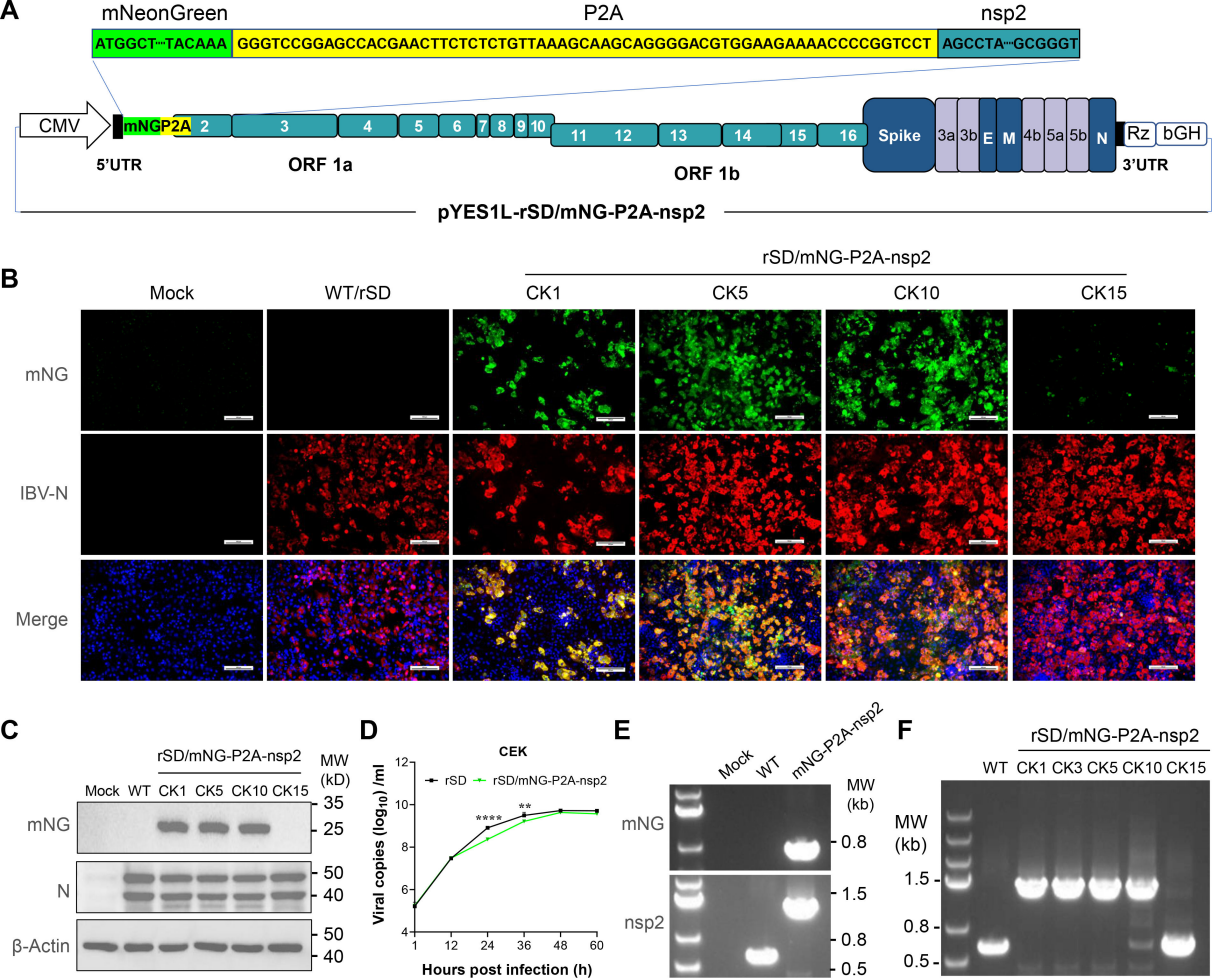
Generation of an rSD expressing mNeonGreen fluorescent protein. (**A**) Schematic representation for the generation of rSD/mNG-P2A-nsp2. The sequence encoding the fusion construct NeonGreen (mNG) was inserted into the viral genome of SD. Insertion of mNG (green) and PTV-1 2A (yellow) between the Viral 5′UTR (black) and nsp2 (dark blue). (**B**) CEK cells were mock infected or infected with WT or rSD/mNG-P2A-nsp2 for 48 h, fixed, and immunostained with an mAb against the viral N protein. Cell nuclei were stained with 4′,6-diamidino-2-phenylindole. Representative images are shown (scale bars, 100 µm). (**C**) Whole cell lysates from CEK cells mock infected or infected with WT or rSD/mNG-P2A-nsp2 for 48 h were subjected to Western blot analysis using antibodies against mNG and the viral N protein. β-actin was used as a loading control. (**D**) Growth kinetics of rSD and rSD/mNG-P2A-nsp2. Cell culture supernatants were collected at specified time points for viral load estimation via PT-qPCR. Data represent the mean ± sd of three independent biological experiments (*n* = 3). Statistical significance was determined by two-sided unpaired Student’s *t*-test without adjustments for multiple comparisons. ****P < 0.0001 and **P = 0.0064. (**E**) Total cellular RNA from CEK cells mock infected or infected with WT or rSD/mNG-P2A-nsp2 was isolated at 48 hpi. RT-PCR was used to amplify mNG (top) or the region between the 5′ UTR and nsp2 (bottom), and the products were separated on a 1% agarose gel. (**F**) Total cellular RNA from CEK cells infected with WT or rSD/mNG-P2A-nsp2 from passages 1, 5, 10, and 15, and gene stability was verified through RT-PCR.

Common reporter genes, such as green fluorescent protein and luciferase, have been integrated into IBV genomes as tracking tools through diverse approaches: (i) direct replacement of accessory proteins (3a/3b and 5a/5b) (27); (ii) intergenic insertion between M and ORF5 genes (28); (iii) C-terminal fusion to structural proteins (S, M, and N) or N-terminal fusion to E protein (29); and (iv) insertion at the nsp13/nsp14 junction leveraging viral protease-mediated self-cleavage (30). To establish a reporter IBV infectious clone, we focused on the first two-thirds of the IBV genome (ORF1), which encodes two polyproteins, pp1a and pp1ab, precursors for 15 nonstructural proteins (nsp2–nsp16). Our innovative strategy employs coupling the mNeonGreen reporter gene with P2A, a self-cleaving peptide derived from the porcine teschovirus-1 (PTV-1) 2A (31), and inserted it upstream of nsps, which allows for bicistronic expression of both endogenous protein (nsp) and mNeonGreen following translation of a single mRNA (Fig. 5A). Using the same strategy, we sequentially inserted mNG-P2A upstream of each nonstructural protein from nsp2 to nsp10 to validate the capacity of polyprotein 1a to accommodate exogenous genes (Table 2).

**TABLE 2:** Tolerance of different sites in the replicase 1a gene of infectious bronchitis virus^*a*^

YAC	Cytopathic effect	RT-PCR	Fluorescence	Recovery of rIBV
mNG-P2A-nsp2	Yes	Yes	Yes	+
mNG-P2A-nsp3	No	No	No	−
mNG-P2A-nsp4	No	No	No	−
mNG-P2A-nsp5	No	No	No	−
mNG-P2A-nsp6	No	No	No	−
mNG-P2A-nsp7	No	No	No	−
mNG-P2A-nsp8	No	No	No	−
mNG-P2A-nsp9	No	No	No	−
mNG-P2A-nsp10	No	No	No	−

^*a*^ +, positive rescue; −, rescue failed.

### Generation and recovery of the reporter virus with mNG-P2A coupled to the nsp2

In this approach, although systematic attempts were made to clone the mNeonGreen reporter gene and P2A element into other sites within the IBV replicase 1a gene, only the recombinant virus with the insertion upstream of IBV nsp2 was successfully rescued (Table 2). We rescued rSD/mNG-P2A-nsp2 virus according to our previously described protocol (Fig. 1). CEK cells infected with rescued virus containing allantoic fluid resulted in the expression of mNeonGreen in the same cells expressing the viral N protein. mNeonGreen was readily detected in whole cell lysate from rSD/mNG-P2A-nsp2, but not rSD/WT−infected CEK cells, while the viral N protein was detected in lysate obtained from both rSD/mNG-P2A-nsp2 and rSD/WT-infected CEK cells (Fig. 5B and C). The multi-step growth kinetics of rSD and rSD/mNG-P2A-nsp2 were evaluated in CEK cells (Fig. 5D). The reporter virus exhibited lower replication than rSD at 24 and 36 hpi. But both viruses showed similar replication levels between 48 and 60 hpi with no significant differences. We confirmed the genetic identity of rSD/mNG-P2A-nsp2 using RT-PCR to amplify the mNeonGreen sequence and the sequence between 5′UTR and nsp2. The mNeonGreen fragment was amplified from cells infected with rSD/mNG-P2A-nsp2, while the fragment between 5′UTR and nsp2 was detected in cells infected with either rSD/WT or rSD/mNG-P2A-nsp2. As predicted, the amplified fragment from rSD/mNG-P2A-nsp2-infected cells had a higher molecular size than the one obtained from rSD/WT-infected cells (Fig. 5E). In the next series of experiments, we examined the genetic stability of rSD/mNG-P2A-nsp2 viruses during serial passage in cultured cells. The recombinant viruses showed complete genetic stability from P1 to P10, as no deletions were detected at the insertion, but loss of mNG expression was complete at P15 (Fig. 5F). To conclude, the nsp2 locus of IBV is a potential site that can accommodate exogenous genes.

## DISCUSSION

Since the QX strain was first reported in Chinese chicken flocks in 1996, numerous IBV strains similar to the QX-like strain have been subsequently identified, becoming one of the dominant epidemic strains in China (6). Chicks have a certain mortality rate, while infected adult hens sustain damage to their reproductive systems, becoming “false-laying hens” unable to lay eggs (32). IBV can infect various tissues and organs, causing significant economic losses to the poultry industry.

Helicases from the model organism *S. cerevisiae* contribute to the regulation of homologous recombination, which is an essential DNA repair pathway for fixing damaged chromosomes (33). Homologous recombination in *S. cerevisiae* has already been used for the generation of a number of molecular virus clones in the past, including members of the *Coronaviridae, Flaviviridae,* and *Pneumoviridae* families (25, 34, 35). When designing or selecting a reverse genetics system, many factors need to be considered, including time required, cost, ease of sequence modification, sequence stability, and laboratory facilities. Unlike conventional reverse genetics techniques such as BAC-based systems or *in vitro* ligation approaches (Table 1), the ingenuity of TAR lies in its capability to divide the IBV genome into seven to eight overlapping fragments and achieve seamless one-step assembly of full-length constructs in *S*. *cerevisia*e. This allows for the random modification of any site, enabling us to effortlessly rescue the rSD strain along with distinct site-specific fluorescent viruses in a single attempt within 1–2 weeks, which is additional proof of the superior cloning efficiency of yeast- versus *E. coli*-based systems. Prior to establishing this platform, our laboratory employed a vaccinia virus-based reverse genetics system for rescuing the YN strain (21), which inherently entailed multiple inefficiencies: (i) cloning target fragments into shuttle plasmids containing selectable markers such as GPT gene, (ii) co-transfecting these constructs into vaccinia virus-infected BHK-21 cells to mediate homologous recombination, (iii) implementing tetracycline-induced positive selection to enrich recombinant vaccinia vectors containing both modified viral sequences and plasmid backbones, and (iv) thymidine kinase-mediated negative selection to excise residual plasmid elements. Furthermore, bulk amplification of recombinant vaccinia stocks followed by ultracentrifugation to purify genomic DNA requires a considerable amount of time. This multi-stage process routinely required 12 weeks for generating a single recombinant virus (13), and its complexity led us to abandon this rescue method.

After yeast assembly, electroporation into *E. coli* is required for further amplification of the plasmid to obtain a sufficient quantity for virus rescue. To prevent plasmid mutations caused by unstable replication in bacteria, we adjusted the culture temperature to 26°C and switched the *E. coli* strain to DH10B. This improvement significantly reduced mutations and eliminated the need for yeast cultivation. The genetic identity of the rescued clone was confirmed by sequencing. Notably, the rSD and rM41 infectious clones replicated in chicken embryos to levels comparable to those of the natural isolate as determined by growth kinetics and pathogenicity.

Reported recombinant viruses similar to clinical isolates have previously been proven to be excellent tools for studying viral infection, replication, pathogenesis, and transmission. The insertion of fluorescent tags or luciferase into different coronaviruses has been shown to be a feasible approach. In studies on mouse hepatitis virus and porcine epidemic diarrhea virus, it has been demonstrated that the nsp2 protein can be replaced or deleted (36, 37). However, the function and replaceability of nsp2 in IBV remain unknown. In coronaviruses, the translation level of proteins encoded by the ORF1a region is higher than that of ORF1b (38, 39). Therefore, we aimed to investigate whether ORF1a can accommodate foreign genes. One important consideration is to minimize the impact of the reporter gene on the viral replication characteristics. We utilized the self-cleaving P2A system to link mNeonGreen fluorescent protein to the viral nsp2 gene, allowing the expression of the fluorescent protein without interfering with nsp2 protein expression. mNeonGreen fluorescent protein is the brightest monomeric green protein yet described and has the notable advantages of superior photostability (40). In previous studies, reporter genes were commonly introduced into coronaviruses by replacing accessory proteins such as 3a, 3b, 5a, or 5b. However, such modifications alter the viral genome and may interfere with viral replication, attenuate virulence, or even hinder successful virus rescue (28, 41). We attempted to insert fluorescence markers into different nonstructural protein (nsp) loci of the viral genome. Regrettably, only the recombinant viruses carrying the insert at the nsp2 position could be successfully rescued. This suggests that nsp2 has a tolerance for exogenous sequence integration, possibly owing to its proximal location within the genome structure. Future studies may adopt alternative strategies to reveal whether other distinct sites in IBV can tolerate foreign sequences. Notably, similar to most reporter viruses, the recombinant construct maintained stable fluorescence expression within 10 passages in cultured cells (28, 31); however, fluorescence was undetectable by the 15th passage, indicating that the reporter gene was gradually lost. This could be due to the inherent exclusion mechanism of the IBV genome, which may also be an issue that RNA viruses cannot avoid. The stability of exogenous genes in recombinant coronaviruses largely depends on the insertion site (27). A previous study demonstrated that a recombinant Beaudette virus carrying a codon-optimized sequence based on IBV codon usage preference exhibited greater genetic stability (28). Future efforts should focus on enhancing replication stability through codon optimization of the fluorescent gene or testing alternative reporter systems to mitigate selection pressure against foreign sequences during prolonged viral propagation.

The homologous recombination mechanism of yeast has overcome the traditional notion that assembling large-genome viruses is challenging. Most BAC or *in vitro* ligation methods are based on modifications of attenuated strains such as Beaudette or H120. However, the TAR platform can accommodate the genetic variability of IBV, enabling the rapid construction of reverse genetics systems for any emerging epidemic strains in a short period. Furthermore, our successful modification within the replicase 1a gene further demonstrates the advantages of this method. In summary, we have developed, for the first time, a powerful, reliable, and convenient IBV reverse genetics system based on the use of TAR. Utilizing this system, we demonstrated the successful accommodation of the mNeonGreen fluorescent reporter gene within the genomic 5′ untranslated region and nsp2 coding sequence, thereby validating the plasticity of this proximal genomic region for exogenous insertions. The stability of the mNeonGreen fluorescent reporter virus allows it to be used for longer-term studies. Thus, TAR technology is also applicable to IBV, providing a rapid and versatile synthetic genomics platform that offers new insights into the molecular biology and pathogenesis of avian infectious bronchitis virus. This strategy offers a huge advantage for the research community and pharmaceutical companies in developing therapeutics for other coronaviruses.

## MATERIALS AND METHODS

### Animals

All SPF ECEs and SPF chickens used in this study were purchased from Beijing Boehringer Ingelheim Vital Biotechnology Co., Ltd. (Beijing, China).

### Cell culture and virus strains

Primary chicken embryo kidney cells (CEK) were prepared from 18-day-old specific-pathogen-free embryos. Baby hamster kidney cells (BHK-21) were cultured in Dulbecco’s modified Eagle’s medium (DMEM, Thermo Fisher Scientific, Waltham, MA, USA) supplemented with 10% fetal bovine serum, 100 units mL^−1^ penicillin, and 100 µg mL^−1^ streptomycin (Gibco, USA). All cells were maintained at 37°C and in a 5% CO_2_ atmosphere. The QX-like strain SD (GenBank: KY421673) and Mass-like strain M41 (GenBank: DQ834384) were preserved in our laboratory and propagated in 10-day-old SPF embryos used as the parental virus in all experiments.

### Assembly of the full length of the IBV genome by TAR cloning in yeast

Based on genomic information of the SD and M41 isolate deposited in GenBank, the full-length genomic sequences were divided into seven fragments (Fig. 1). For the initial construction, it is best to clone the seven fragments into the high-copy-number pUC57 plasmid to ensure a single template without additional mutations and facilitate subsequent modifications of individual fragments. The CMV promoter was amplified and fused to the F1 fragment containing the 5′ UTR of the genome. Meanwhile, an HDV ribozyme sequence and a BGH terminator sequence were fused together as a separate F8 fragment. Each fragment has at least a 40 bp overlap with the adjacent fragments. Each DNA fragment was amplified by PCR using the CloneAmp HiFi PCR premix (Takara). PCR primer pairs used to amplify genomic regions are listed in Table S1. The amplification conditions were as follows: denaturation at 98°C for 10 s, annealing for 5 s at a primer-dependent temperature (53°C–60°C), extension at 5 s/kb, for a total of 30 cycles. The PCR products were purified using the E.Z.N.A. Gel Extraction Kit (Omega Bio-tek, USA, No. D2500-02). Two hundred nanograms of F1-F8 was co-transformed with 100 ng of the linearized BAC/YAC shuttle pYES1L vector (Thermo Fisher Scientific, USA) into *Saccharomyces cerevisiae* strains Mav203, VL6-48, and W303, purchased from Zoman Biotechnology Co., Ltd. (Beijing, China), using the lithium acetate method and incubated at 30°C for 48 h to obtain single colonies. Individual colonies were resuspended in 15 µL lysis buffer (1 M sorbitol, 50 mM Tris-HCl [pH 7.5], 10 mM EDTA, and 1% [wt/vol] SDS preheated to 50°C). After a 30 min incubation at room temperature, only 5 µL of lysate was taken and heat inactivated at 95°C for 5 min, serving as a template (≤0.5 µL) for colony PCR (note: residual SDS in lysates may inhibit PCR efficiency). PCR-confirmed clones (1 µL unheated lysate) were electroporated into *E. coli* strains (DH10B or EPI400; Zoman Biotechnology Co., Ltd., Beijing, China) using an electroporator (Bio-Rad) under the following conditions: 1.8 kV, 200 Ω, and 25 µF. Transformed competent cells were cultured on LB agar at 26°C and 30°C for 48 h to select single colonies. Positive clones were inoculated into LB broth for plasmid extraction (NucleoBond Xtra Midi Kit, MACHEREY-NAGEL). Construct integrity was verified through Sanger sequencing (Tsingke, Nanjing, China). Full-genome mutational screening was performed via Illumina-based deep sequencing. To construct an infectious cDNA clone of rSD/mNG-P2A-nsp2, engineering efforts were exclusively confined to fragment F1. Then, they remained consistent with the aforementioned protocols for the parental IBV reverse genetics platform.

### Recovery of infectious virus

To recover infectious virus, shuttle plasmid (pYES1L-rIBV BAC/YAC) and helper plasmid (pCMV-IBV-N) were cotransfected into BHK-21 cells using Lipofectamine 2000 (Thermo Fisher Scientific). Briefly, 3 µg of pYES1L-rIBV BAC/YAC DNA and 0.5 µg of pCMV-IBV-N plasmid were diluted in 250 µL of Opti-MEM (Thermo Fisher Scientific). A volume of 7 µL Lipofectamine 2000 was diluted in 250 µL Opti-MEM and incubated for 5 min at room temperature. The diluted DNA was combined with diluted Lipofectamine 2000, incubated for 20 min at room temperature, and added dropwise to confluent BHK21 cells grown in a 6-well plate. After incubation at 37°C for 5 h, the transfection medium was exchanged for post-infection medium (DMEM supplemented with 2% [vol/vol] FBS).

After 48 h, BHK-21 cell lysates, obtained through three cycles of freeze thaw (−80°C/37°C alternation), were clarified by centrifugation (5,000 × *g*, 3 min). Subsequently, 500 µL supernatant per sample was inoculated into the allantoic cavity of 10-day-old SPF embryonated chicken eggs. The first-passage allantoic fluid virus was inoculated into 10-day-old specific pathogen-free embryonated chicken eggs to observe pathogenicity at 144 h post-inoculation or into primary CEK cells for indirect immunofluorescence assay to detect viral antigens, with successful virus rescue confirmed by quantitative reverse transcription PCR targeting viral genomic RNA.

### Indirect immunofluorescence assay

Infected CEK cells grown in 12-well plates were fixed with 4% formaldehyde in phosphate-buffered saline (PBS) for 20 min, permeabilized with 0.1% Triton X-100 in PBS for 10 min, and blocked with 5% (wt/vol) non-fat milk in PBS for 30 min. Anti-IBV nucleocapsid (N) monoclonal antibody (mAb) (diluted 1:1,000 in PBS, Hytest, Turku, Finland) was applied to 12-well plates and incubated overnight at 4°C. After three PBS washes, goat anti-mouse IgG conjugated with Alexa Fluor 555 fluorophores (CST, #8953) diluted 1:1,000 in PBS was used as a secondary antibody. Afterward, the 12-well plates were washed three times with PBS. Following nuclear counterstaining with 4′,6-diamidino-2-phenylindole (CST, #4083) for 5 min at room temperature, fluorescence imaging was performed using a Nikon camera.

### Growth kinetics evaluation of infectious recombinant IBVs

Virus growth kinetics were analyzed in both CEK cells and ECEs. The CEK cells were infected with rSD/mNG-P2A-nsp2 at an MOI of 0.01. After 1 h of adsorption, surface virions were removed, and the cells were washed three times with PBS. For assessing the replication curves of rSD and rM41 in embryonated chicken eggs, 10-day-old embryos were inoculated with 10⁴ viral genome copies, and cell supernatants or allantoic fluids were collected at specified time points (1, 12, 24, 36, 48, and 72 h post-inoculation). Viral RNA was extracted by Hipure RNA minikit (Magen, Beijing, China), reverse-transcribed by M5 Super qPCR RT kit (Mei5bio, Beijing, China), and subjected to quantitative real-time PCR on Light Cycler 96 using 2ⅹM5 Hiper SYBR Premix EsTag (Mei5bio, Beijing, China). Primers were designed based on the conserved 5′ UTR of IBV (F:5′-GTTGGGCTACGTTCTCGC-3′, R:5′-AAGCCATGTTGTCACTGTCTAT-3′) to determine viral particle copy numbers for constructing replication kinetic curves. Copy number (×) for each qPCR reaction was calculated from its Ct value using the previously established curve (Ct = −3.41 × log_10_^x^ + 39.1).

### Western blotting

To examine mNG expression, CEK cells were infected with the rSD/ mNG-P2A-nsp2 viruses at an MOI of 0.01. At 48 h post-infection, the culture supernatant was discarded, followed by three washes with ice-cold PBS. Cells were lysed on ice for 15 min using radioimmunoprecipitation assay buffer containing protease inhibitors, then centrifuged at 12,000 × *g* for 5 min. The supernatant was collected, mixed with loading buffer, and boiled for 10 min. For each lane, 15 µg of protein was loaded and separated by SDS-PAGE, followed by transfer of denatured proteins onto a PVDF membrane. The membrane was blocked with 5% skim milk at room temperature for 1 h, washed three times with TBST, and incubated overnight at 4°C with IBV-N monoclonal antibody (diluted 1:1,000 in PBS, Hytest, Turku, Finland) and anti-mNeonGreen Tag antibody (diluted 1:1,000 in PBS, CST, USA). After three additional TBST washes, the membrane was incubated with HRP-conjugated goat anti-mouse secondary antibody (diluted 1:3,000 in PBS, CST, USA) at room temperature for 1 h. Finally, the ECL substrate was applied, and the membrane was imaged using a multifunctional imaging system (ChemiDoc MP, Bio-Rad).

### mNeonGreen reporter gene stability assay

To investigate the stability of the mNG fluorescent protein and determine how many generations the mNG reporter gene can be maintained in the IBV genome, the rescued virus strain rSD/mNG-P2A-nsp2 was inoculated into CEK cells at an MOI of 0.01. The culture supernatant was harvested every 48 h, counted as one passage. For each new passage, 100 µL of the previous supernatant was absorbed onto CEK cells for 1 h. The inoculum was then removed, and the cells were cultured in DMEM with 2% FBS. This was repeated for 15 passages. Primers targeting regions flanking the nsp2 gene (forward: GTTGGGCTACGTTCTCGC, reverse: GTGTAGAAAAACAAAGCGTCAC) were designed for PCR analysis. The expected product size is 230 bp for the wild-type sequence, while insertion of the mNG reporter increases the fragment length by 711 bp (941 bp total). Genomic RNA was extracted every five passages and subjected to PCR to confirm the integrity of the mNG insertion.

### Statistical analysis

Statistical analyses were done using the GraphPad Prism 8.0.1 software. All applied statistical tests can be found in the respective figure legends. If *P* ≤ 0.05, the results were considered significantly different. All experiments in which statistical tests were used were repeated independently at least three times.

## Data Availability

The authors confirm that all data underlying the findings are fully available without restriction. All relevant data are within the paper and its supplemental material.

## SUPPLEMENTAL MATERIAL

Table S1 jvi.00535-25-s0001.docx Primers used in the study.

ASM does not own the copyrights to Supplemental Material that may be linked to, or accessed through, an article. The authors have granted ASM a non-exclusive, world-wide license to publish the Supplemental Material files. Please contact the corresponding author directly for reuse.
