# Supplementary material for: Rapid reconstruction of infectious bronchitis virus expressing fluorescent protein from its nsp2 gene based on transformation-associated recombination platform
Source: J Virol. 2025 Jun 5;99(7):e00535-25. doi: 10.1128/jvi.00535-25 (PMC12282139; doi:10.1128/jvi.00535-25)
Supplement: Table S1 — Primers used in the study. [file jvi.00535-25-s0001.docx]

Table S1. Primers used in the study.

| **Primer Name** | **Sequences (5’-3’)** | **Purpose** |
| --- | --- | --- |
| pYES1L-F | CCTCGCCGCAGTTAATTAAAGTCAGTG | Vector amplification |
| pYES1L-R | CGGCGGTATCAGCGCGGCCG |  |
| CMV-F | GAACGACCGAGCGCAGCGGCGGCCGCGCTGATACCGCCGCGACATTGATTATTGACTAG | CMV promoter amplification |
| CMV-R | ACGGTTCACTAAACCAGCTCTGC |  |
| rSD-F1 | TCTATATAAGCAGAGCTGGTTTAGTGAACCGTACTGAAGATAGATATTATTAT | SD genome amplification |
| rSD-R1 | TCTTTTTCTTGAGGCACAAATTT |  |
| rSD-F2 | CTGATGTTCCTACAGAAAAATT |  |
| rSD-R2 | CAACTCTTAAACCAACTAACAG |  |
| rSD-F3 | GTGGTGTTGTTAGTAGCACTGT |  |
| rSD-R3 | ATCCATGACCTATCCAACACTGAC |  |
| rSD-F4 | TGTCTACGTAATAAGGTTTGCAC |  |
| rSD-R4 | ACCTCATCTACCAACAGAATGTC |  |
| rSD-F5 | GCCAGAAGTTAGTTGTGACATTC |  |
| rSD-R5 | TGCACTACATAGTGCACACAAAAT |  |
| rSD-F6 | ACGGCAAGTTATTGATTAGAGATG |  |
| rSD-R6 | TTTGAACCAT**C**AAACAGACTTTTT**G**GGTCTGTATTGTTCAGTCACCACAT |  |
| rSD-F7 | CAGACC**C**AAAAAGTCTGTTT**G**ATGGTTCAAACTCCCG |  |
| rSD-R7 | CGACCCTTTTTTTTTTTTTTTTTTTTTTTTTGCTCTAACTCT |  |
| HDVr+BGH-F8 | AGAGCAAAAAAAAAAAAAAAAAAAAAAAAAGGGTCGGCATGGCATCTCCACC | Hepatitis delta virus ribozyme and BGH amplification |
| HDVr+BGH-R8 | GCGCTTCCTCGCTCACTGACTTTAATTAACTGCGGCGAGGCCATAGAGCCCACCGCATCCCCAGCATGCCTGCTATTGTCTT |  |
| 5’UTR-tail-R | TCTTCTTCTCCCTTGCTGACTGAAGCCATGTTGTCACTGTCTAT | mNG gene insertion |
| mNG-P2A-F | CAGTGACAACATGGCTTCAGTCAGCAAGGGAGAAGAAGACAA |  |
| mNG-P2A -R | AGGACCGGGGTTTTCTTCCACGTCCCCTGCTTGCTTTAACAGAGAGAAGTTCGTGGCTCCGGACCCTTTGTAAAGCTCATCCATTCCCA |  |
| P2A-nsp2-F | GTTAAAGCAAGCAGGGGACGTGGAAGAAAACCCCGGTCCTAGCCTAAAACAGGGAGCATCTTCCCAA |  |
| rSD-J1-F | TATAGGATACCGCTAGGGGCGCTG | Verification of SD recombination |
| rSD-J1-R | AATAATATCTATCTTCAGTAC |  |
| rSD-J2-F | GTTGTTGATGTCCTTGGAGAT |  |
| rSD-J2-R | CTGCTTACAAGTTGCGTCG |  |
| rSD-J3-F | CGCCAGTAGTGTGGAAGTAT |  |
| rSD-J3-R | TTGTTGAGGCACAATAAGC |  |
| rSD-J4-F | GGTATGAAGGTGGGTGAT |  |
| rSD-J4-R | TCTATTGGATCGTACCAATC |  |
| rSD-J5-F | TAATGTAATGGTACCGGAGTGT |  |
| rSD-J5-R | GTATACCACGCTTAGCTCTT |  |
| rSD-J6-F | GAGCTGGAAGTGATAAAG |  |
| rSD-J6-R | CTACATAGTGCACACAAAAT |  |
| rSD-J7-F | TTGTTGTGGATGCTTTGGT |  |
| rSD-J7-R | ACCGTTCTTCGGGAACTC |  |
| rSD-J8-F | CAGCACTAGGACGCCCATT |  |
| rSD-J8-R | ACCGCATCAGGATTCGCTACCT |  |
| rM41-F1 | TATATAAGCAGAGCTGGTTTAGTGAACCGTACTTAAGATAGATATTAAT | M41 genome amplification |
| rM41-R1 | CCAAAGGTGACAGTTTTACC |  |
| rM41-F2 | ATGTGGTTTGCAAAGCAGG |  |
| rM41-R2 | AAAGTGTTATTAATAACACCACCT |  |
| rM41-F3 | TGGTAGAGAAAAAGGCAGG |  |
| rM41-R3 | GAAAACTCTTGAGTAACCGAT |  |
| rM41-F4 | AGAGGTCAACTGTATTACAATC |  |
| rM41-R4 | ACTACACAAACGCCACAAG |  |
| rM41-F5 | GAGCCCCTACAACATTAC |  |
| rM41-R5 | TAAGCAATATTGTCGATAGACT |  |
| rM41-F6 | AAAGTTTTTCAGCTCTCCAG |  |
| rM41-R6 | AAGCAGCAATTTTTTCTTGATTC |  |
| rM41-F7 | CAGTCACTTTTGTTGAAGAATC |  |
| rM41-R7 | CGACCCTTTTTTTTTTTTTTTTTTTTTTTTTGCTCTAACTCTATACTAGCCT |  |
| rM41-J1-F | TATAGGATACCGCTAGGGGCGCTG | Verification of M41 recombination |
| rM41-J1-R | GCAAGGCTAGTGTGATAGAT |  |
| rM41-J2-F | TGTTTCAATCAGCGCGTGTG |  |
| rM41-J2-R | CACAACATCGAGAGCGTCCT |  |
| rM41-J3-F | ATAACAAAGTCTGGTGCTAAAC |  |
| rM41-J3-R | AGTAGAACCACAGAAAACGC |  |
| rM41-J4-F | TGCATGCTTATCTTGTTGAGT |  |
| rM41-J4-R | GTTACACGCGCCTCTTTATA |  |
| rM41-J5-F | AGCAGGTTTATAGGCGAGT |  |
| rM41-J5-R | TTCTAGTAAAGTGAAAGCCAG |  |
| rM41-J6-F | CATGTGTTGATGCTCTTAAAG |  |
| rM41-J6-R | CGACCCTGTGTGTTTAATGT |  |
| rM41-J7-F | TGTTAACAACTCCTAGTAGTC |  |
| rM41-J7-R | CACTCTAATATGCTCCGC |  |
| rM41-J8-F | GTCGCAGGAGTGGTTCTGAA |  |
| rM41-J8-R | ACCGCATCAGGATTCGCTACCT |  |

F: forward primer; R: reverse primer. J: Junction. Homologous arms are indicated by underlines, and molecular markers are shown in red text.
